# Partnership working between the Fire Service and NHS: delivering a cost-saving service to improve the safety of high-risk people

**DOI:** 10.1186/s13104-015-1120-1

**Published:** 2015-04-14

**Authors:** Joyce A Craig, Shelagh Creegan, Martin Tait, Donna Dolan

**Affiliations:** Craig Health Economics Consultancy, 31 Belmont Drive, Giffnock Glasgow, G46 7NZ UK; Associate AHP Director Mental Health and Learning Disabilities, NHS Tayside, Dundonald Centre, Unit 9a, Manhattan Works, Dundonald Street, Dundee, DD3 7PY UK; Group Manager (Dundee), Scottish Fire & Rescue Service, Macalpine Road, Dundee, DD3 8SA UK; Firefighter (Control), Dundee Operations Control Red Watch, Scottish Fire and Rescue Service, Macalpine Road, Dundee, DD3 8SA UK

**Keywords:** Economics, Fire risk assessment, Partnership, NHS, Fire service

## Abstract

**Background:**

The Scottish Fire and Rescue Service and NHS Tayside piloted partnership working. A Community Fire Safety Link Worker provided Risk Assessments to adults, identified by community health teams, at high risk of fires, with the aim of reducing fires. An existing evaluation shows the Service developed a culture of ‘high trust’ between partners and had high client satisfaction. This paper reports on an economic evaluation of the costs and benefits of the Link Worker role.

**Methods:**

An economic evaluation of the costs and benefits of the Link Worker role was undertaken. Changes in the Risk Assessment score following delivery of the Service were used to estimate the potential fires avoided. These were valued using a national cost of a fire. The estimated cost of delivering the Service was deducted from these savings.

**Results:**

The pilot was estimated to save 4.4 fires, equivalent to £286 per client. The estimated cost of delivering the Service was £55 per client, giving net savings of £231 per client. The pilot was cost-saving under all scenarios, with results sensitive to the probability of a fire.

**Conclusions:**

We believe this is the first evaluation of Fire Safety Risk Assessments. Partnership working, delivering joint Risk Assessments in the homes of people at high risk of fire, is modelled to be cost saving. Uncertainties in data and small sample are key limitations. Further research is required into the *ex ante* risk of fire by risk category. Despite these limitations, potential savings identified in this study supports greater adoption of this partnership initiative.

## Background

A key action for allied health professionals is strengthening partnerships with outside agencies [1]: an aim mirrored in the Scottish Fire and Rescue Service (SFRS) which regards partnership working as essential to improving fire prevention and hence the safety and wellbeing of people [2].

In Spring 2012, SFRS and NHS Tayside recruited a Community Fire Safety Link Worker to provide joint Fire Safety Risk Assessments in the homes of adults at high-risk of domestic fires (‘the Service’) and develop effective partnership working with a range of agencies, including healthcare teams. The aim is to reduce occurrence of fire, resultant injury, loss of life and property damage, for those at high-risk due to physical and mental health needs.

Initially, the Link Worker delivered awareness-raising sessions on fire risks and the benefit of preventative strategies to community health teams managing clients with mental health disorders or vulnerable older people within Dundee, a city in Scotland. Many clients pose multiple risks for accidental dwelling fires being smokers, users of alcohol or drugs, having poor mental health, or having disabilities or infirmities. [3]. Many are also socially isolated and unable to access sources of fire prevention advice.

Fire Safety Risk Assessments are usually initiated by an NHS community healthcare practitioner, trained on fire safety risk factors, who refers potential clients to the Link Worker. Following the client’s agreement, a joint Fire Safety Risk Assessment is undertaken by the Link Worker and a healthcare practitioner. The Link Worker provides information to reduce the risk of fire and advise on fire exits, providing where necessary, equipment such as smoke-alarms and fire-resistant bedding. Feedback is provided to the referring team and to Fire Service operational personnel to assist with future incidents.

The Risk Assessment includes completing a Home Fire Service Risk Rating Form [4] before and after the Service is provided. This form is widely used by Fire Services and has 17 questions identifying fire risk of occupants (e.g. age, mobility, smoking status, knowledge of fire safety) and presence of working smoke alarms and fire escape plan. Each response is scored and the total determines if occupants are at high, medium or low risk of fire. Change in fire risk status is a key outcome of the pilot.

A pilot was undertaken during which 138 clients received joint Risk Assessment home visits. Of 138 visits, 87 were conducted jointly with community NHS teams, 50 with a second person from the Fire Service and no data were available on one visit. A recent evaluation [5] showed that, following the Link Worker’s intervention, the number of clients at high-risk of fire fell from 54% to 39%; those at medium-risk fell from 30% to 5%, whilst those at low-risk increased from 16% to 56%.

### Economic evaluation

This paper provides an economic evaluation of the Link Worker role to inform decisions on its wider adoption. The research question is: *Is the Link Worker service cost effective in terms of the number of potential fires avoided?*

### Data

Structured literature searches were undertaken to identify existing evaluations of fire Risk Assessments and cost of domestic dwelling fires. These were supported by hand searching, primarily of local fire service and local authority documents, including those related to the pilot, to provide data for modelling. Interviews were conducted with the Link Worker, an NHS IT lead and NHS community mental health practitioner to gather information about resources and costs of the Service.

No existing evaluation was identified thus de novo modelling was undertaken in Excel®. The data informing the model are now described.

### Dwellings and fire risks

At December 2013, there were 73,901 residential properties in Dundee [6]. During 2012/13 there were 269 dwelling fires attended by SFRS, giving a fire-risk of 0.364%.

In 2012/13, fire crews undertook 4,641 Risk Assessments in Dundee, using the same Risk Rating Form (4) and scored 80% of homes at low-risk of fire, 16% at medium-risk and 4% at high-risk. This is judged to provide an unbiased risk profile of all dwellings in Dundee.

No data on the fire-risk associated with each risk category were available from the literature or SFRS records. However, there is substantial evidence that social deprivation is related to increased fire risk [3]. Moreover, the deprivation status for each of the 269 properties experiencing a fire in Dundee in 2012 could be estimated using a look-up table that maps postcodes to the Scottish Index of Multiple Deprivation (SIMD) [7]. Undertaking the mapping confirmed a strong relationship between number of fires and socioeconomic grouping. Over 56% (151) of fires were in properties in areas with the lowest 20% SIMD ranking; 28% (74) fires were in areas in the SIMD group 21% to 50% and 16% (44 fires) occurred in properties with a SIMD score of over 50%.

### Cost of domestic fires

The estimated average cost of a domestic dwelling fire in 2004 was £24,900, of which £14,600 was related to injuries and fatalities, £7,300 for property damage and £3,000 for response and other costs [8]. Costs were updated using data from Department of Transport [9] for injuries and death and Consumer Price Index for remaining values, to give a cost of £32,390 per domestic dwelling fire in 2013 prices.

### Estimated cost of service

Records completed by the Link Worker show that mean time per client was 103 minutes. The average hourly cost was £23.91 including overheads, giving a staff cost per client of £41.03. Travel costs were estimated at £5.00 for an average return journey of 10 miles. The mean cost per client to provide fire retardant bedding, smoke alarms etc. was £5.42.

NHS Tayside staff required 10 minutes for additional administrative tasks, equivalent to £3.50 per client. IT developments costs were under £550, equivalent to £0.35 per client assuming a 3 year life. Hence total estimated cost per client is £55.31 (2013 prices).

This assumes a joint Risk Assessment is conducted by an NHS team member and the Link Worker, with no additional cost for the NHS member who would be visiting the client anyway. However, for 50 (35%) visits the Link Worker was accompanied by a second SFRS staff member, not an NHS member. The additional cost is estimated at £32.71 per client (78 minutes per client at cost of £25.17 per hour), giving a total cost of the Service of £88.02 per client.

## Methods

The main outcome is net cost or saving per client receiving a joint Fire Safety Risk Assessment. The methodology used to calculate that value is described and used the following steps:

- Quantify the potential number of dwelling fires avoided as a result of the Service;
- Value savings from averted fires and express as saving per client;
- Deduct costs to deliver the Service
- Undertake sensitivity analyses.

Table 1 provides a worked example of these steps, assuming 500 clients per year receive Fire Safety Risk Assessments. This value is informed by the referral rate during the pilot.

**Table 1:** **Worked example of steps in economic evaluation**

**Step**	**Low fire-risk**	**Medium fire-risk**	**High fire-risk**	**Total**
A. Allocate dwellings across Dundee by risk reported from 4,641 Risk Assessments	58,933 (80%)	11,990 (16%)	2,978 (4%)	73,901
B. Number of fires from SIMD analysis of postcodes	44	74	151	269
C. Probability of fire (B/A)	0.075%	0.617%	5.071%	0.364%
D. Allocate 500 dwellings using fire risk pre Service	78 (16%)	153 (30%)	269 (54%)	500
E. Estimate number of fires (C*D)	0.06	0.94	13.62	14.63
F. Allocate 500 dwellings using fire risk post Service	280 (56%)	26 (5%)	194 (39%)	500
G. Estimate number of fires post-Service (F*C)	0.21	0.16	9.84	10.21
H. Estimate fires saved (E-G)	- 0.15	0.78	3.78	4.42
I. Savings (H* £32,390)	-£4873	£25360	£122573	£143061
J. Saving per client I/500				£286
K. Cost per client				£55
L. Net savings per client (J-K)				£231

Note values are rounded to nearest pound

Steps A to C calculate the fire-risk for properties classified as low, medium and high across Dundee. At Step A, the 73,901 residential dwellings are allocated across risk categories using data from the 4,641 Risk Assessments conducted by fire crews. The 269 fires are allocated to risk categories according to the SIMD (Step B). The resulting probability of a fire by risk category is calculated (Step C).

Steps D and E estimate the probable number of fires in the 500 dwellings visited by the Link Worker given the initial fire categories (low, medium, high) multiplied by probability of a fire in each category. Steps F and G estimate the probable number of fires given the updated fire categories (low, medium, high) following the Link Worker’s input, multiplied by the probability of a fire in each category.

The estimated fires saved as a result of the Service is calculated at Step H. The associated savings are estimated by multiplying fires saved by the cost of a dwelling fire (Step I) to give gross savings. These are expressed as a saving per client at Step J. Cost of the Service is deducted from gross savings at Step L to give net savings per client. In this example, the potential number of fires before the Risk Assessment is 14.6 fires, falling to 10.2 afterwards, saving 4.4 fires, equivalent to a financial saving of £143,061 or £286 per client visited. The Service-related cost per visit of £55 is deducted to give net savings of £231 per client visited.

## Results

Table 2 reports the central case results showing fires saved, gross savings, cost of visits and net savings per client. Sensitivity analysis show the savings associated with:

- A 25% lower saving per dwelling fire avoided of £24,290; and
- Higher cost assuming two SFRS staff conduct Risk Assessment visit.

**Table 2:** **Results for central case and sensitivity analyses per client**

**Scenario**	**Gross savings**	**Cost of visits**	**Net savings**
Central case: 4.4 fires averted, £32,390 saving per fire averted, 1 SFRS & 1 NHS employee	£286	£55	£231
With 2 SFRS employees	£286	£88	£198
With lower savings per fire avoided (£24,290)	£215	£55	£160
2 SFRS staff & £24,290 per fire avoided	£215	£88	£127

Further sensitivity analyses are provided assuming a 5% change in the annual number of fires and using a different allocation of fires to SIMD categories.

Under the central case, savings per joint Risk Assessment conducted are £231. If the lower cost of an avoided fire is adopted (£24,290), savings reduce to £160 per client. For Risk Assessments carried out by two SFRS personnel, the estimated savings are £198, falling to £127 with the lower savings per fire avoided.

Assuming 5% more fires, 282 fires per annum compared to 269 in the central case, absolute risk of a fire across all dwellings increases to 0.382% (282/73901). Assuming joint NHS and SFRS Risk Assessments are conducted and a saving per fire avoided of £32,390, savings per client increase to £245 from £231, reflecting the higher fire risk and hence higher benefit from avoided fires. Applying a lower saving from fires avoided (£24,290) reduces savings to £170 per client. Conducting Risk Assessments using two SFRS personnel reduces savings to £212 and £137 per client respectively.

Assuming 5% fewer fires, (256 compared to 269), absolute risk of a fire across all dwellings decreases to 0.346% (256/73,901). With joint NHS and SFRS Risk Assessments and a saving per fire avoided of £32,390, savings per client reduce to £217, reflecting the lower fire risk and hence lower benefit from avoided fires. Applying a lower saving from fires avoided (£24,290) reduces savings to £149 per client. Conducting Risk Assessments using two SFRS personnel reduces savings to £184 and £116 per client respectively.

Allocating fires in SIMD categories such that all fires in the first third of SIMD categories are classed high-risk, those in the next third of SIMD categories are classed as medium-risk and the remaining third as low-risk allocates 72% of fires to a high-risk category, 17% to medium and 11% to low-risk. This increases the probability of a fire in high-risk properties and hence increases the potential benefit from Service. Assuming joint NHS and SFRS Risk Assessments and a saving per fire avoided of £32,390, savings per client increase to £282, reflecting the higher fire-risk avoided. Applying a lower saving from fires avoided (£24,290) reduces savings to £198 per client. Conducting Risk Assessments using two SFRS personnel reduces savings to £249 and £165 per client respectively.

These analyses indicate results are most sensitive to the *ex ante* risk of a fire in high-risk dwellings. In all cases the Service is cost saving, with the lowest savings being around £100 per client.

## Discussion

This potential benefit of joint partnership working to deliver effective fire prevention services is highly relevant to Scotland. Despite a steady decline in the number of fires and fire casualties, Scottish rates remain about 40 per cent above the levels elsewhere within the UK [10]. Higher rates of deprivation, smoking and alcohol abuse are important risk factors for fire [3] and similar risk factors apply to clients of NHS community mental health teams. Cross-referring clients, using clear protocols and efficient information-sharing systems, combined with the effective delivery of well-validated Risk Assessments has been demonstrated to enhance the safety of high-risk individuals and deliver net savings for society.

We believe this is the first evaluation to link change in risk category following a Risk Assessment visit to potential savings from fires averted. Audit Scotland identified a paucity of evidence on the impact of preventative services [10]. Rather Fire and Rescue Services have correlated the long-term fall in fires and related casualties with prevention programmes, without adjusting for other national trends such as decline in smoking rates and increased use of fire-retardant upholstery [10].

Potential limitations with the economic evaluation relate to uncertainties with data, including the cost of domestic fires. More research is required to establish the mean cost of fire in homes occupied by high-risk groups. Sensitivity analysis indicate the results are most sensitive to the *ex ante* risk of a fire in dwellings classified as low, medium and high risk. These probabilities cannot be observed directly and deprivation status has been used as a proxy measure. Further research in this area would improve confidence in the results.

Data collected by the SFRS from the 4,641 Risk Assessments are a potential source of bias, particularly if dwellings at low-risk of fire are more likely to be visited than other dwellings. The Risk Assessment scale is designed to identify fire hazards and people at risk and to remove or reduce the risk of those hazards causing harm to as low as is reasonably practicable. Its goal is to reduce the probability of a fire starting. However, no validation of its use in categorising dwellings into risk categories has been identified and it may have poor sensitivity.

The pilot was small with only 138 clients. It is not known if these clients are similar to those who may be identified as suitable for a fire Risk Assessment if the Service was rolled-out more widely.

## Conclusions

This economic evaluation indicates partnership working between the SFRS and the NHS to provide joint Fire Safety Risk Assessments for vulnerable people is likely to be cost saving. Despite limitations related to quality of evidence and concerns about generalising from a pilot to a bigger programme, the potential cost savings of over £230 per Risk Assessment support more widespread adoption, with continued evaluation, of this partnership initiative to improve community fire – prevention for vulnerable people.

### Ethics

This study does not require ethical approval by an ethics committee. Informed consent was gained from all clients prior to a joint Risk Assessment visit.

## Abbreviations

MiDIS: Multi Disciplinary Information System
SFRS: Scottish Fire and Rescue Service
SIMD: Scottish Index of Multiple Deprivation
